# Association of handgrip strength with hospitalization, cardiovascular events, and mortality in Japanese patients with type 2 diabetes

**DOI:** 10.1038/s41598-017-07438-8

**Published:** 2017-08-01

**Authors:** Hidetaka Hamasaki, Yu Kawashima, Hisayuki Katsuyama, Akahito Sako, Atsushi Goto, Hidekatsu Yanai

**Affiliations:** 1 Department of Internal Medicine, National Center for Global Health and Medicine Kohnodai Hospital, 1-7-1 Kohnodai, Ichikawa, Chiba, 272-8516 Japan; 2 Hamasaki Clinic, 2-21-4, Nishida, Kagoshima, 890-0046 Japan; 3 Institute for Clinical Diabetology, German Diabetes Center, Leibniz Center for Diabetes Research, Heinrich Heine University, Auf’m Hennekamp 65, Düsseldorf, 40225 Germany; 4 Epidemiology and Prevention Group, Center for Public Health Sciences, National Cancer Center, 5-1-1 Tsukiji, Chuo-ku, Tokyo, 104-0045 Japan

## Abstract

Handgrip strength is useful for the diagnosis of sarcopenia. We examined the associations of handgrip strength with all-cause mortality, cardiovascular events, and hospitalization in patients with type 2 diabetes. From April 2013 to December 2015, we conducted a retrospective cohort study to examine patients with type 2 diabetes whose handgrip strength was measured at our hospital. All patients were followed up until May 2016. A total of 1,282 patients (63.8 ± 13.9 years) were enrolled and followed up for 2.36 ± 0.73 years. During the follow-up period, 20 patients (1.6%) died, 14 (1.1%) experienced cardiovascular events, and 556 (43.4%) were admitted to our hospital for any diseases. Multiple regression analyses revealed that handgrip strength was favorably associated with abdominal obesity and renal function. Moreover, Cox proportional hazard analyses with adjustment for potential confounding variables revealed that handgrip strength was significantly associated with occurrence of CVD events and hospitalization in all subjects. In addition, handgrip strength was significantly associated with mortality and hospitalization in men and with hospitalization in women. Handgrip strength could be a prognostic indicator for health as well as a diagnostic marker of skeletal muscle mass loss in Japanese patients with type 2 diabetes.

## Introduction

Sarcopenia, which is defined as age-related loss of muscle mass, strength, and function^1^, has become a serious problem in aging societies. Sarcopenia has been independently associated with physical disability and functional impairment^2^ and mortality^3^. From 1990 to 2013, diabetes has been one of the top 20 disease and has consistently contributed to global burden of disease and disability-adjusted life years^4^. Patients with type 2 diabetes show lower energy expenditure, duration of physical activity^5^, cardiorespiratory fitness^6, 7^, and muscle strength^8, 9^ than those of non-diabetic subjects. Type 2 diabetes has been shown to be associated with skeletal muscle loss in community-dwelling older individuals^10^. Diabetic neuropathy is also associated with muscle weakness. Patients with diabetic sensorimotor polyneuropathy have significantly lower knee extensor strength than those without the disease^11^. Resistance training can significantly improve the speed of ankle and knee strength generation in patients with diabetic neuropathy compared with patients without diabetes^12^. Longitudinal studies have shown that muscle weakness evaluated by handgrip strength is associated with the risk of incident diabetes in older individuals^13, 14^. Furthermore, sarcopenic obesity (combination of muscle mass decline and excess adiposity)^15^, which is common in older patients with type 2 diabetes, increases all-cause mortality^16^. Hence, prevention of sarcopenia is important in the management of type 2 diabetes.

Handgrip strength is a simple and cost-effective method for evaluating overall muscle strength and is useful for the diagnosis of sarcopenia^17^. Previous studies have shown a significant relationship between handgrip strength and mortality in young, middle-aged, and older individuals^18–20^. Leong *et al*.^21^ recently reported that handgrip strength was negatively associated with all-cause mortality, cardiovascular mortality, myocardial infarction, and stroke, whereas no significant associations of handgrip strength with the incidence of diabetes and risk of hospital admission for chronic obstructive pulmonary disease, pneumonia and respiratory illness were observed. This large, longitudinal, international, joint study also suggested that handgrip strength is a useful predictor for the risk of cardiovascular disease (CVD) and mortality. However, to date, there have been few studies that have investigated the association between handgrip strength and prognosis in a type 2 diabetic population. Thus, this study aims to examine the associations of handgrip strength with mortality, CVD events, and hospitalization in patients with type 2 diabetes.

## Results

This study enrolled 1,282 patients (709 men and 573 women) with type 2 diabetes. The mean age of study participants was 63.8 ± 13.9 years. The mean body mass index (BMI) was 25.5 ± 5.4 kg/m^2^. The patients’ mean duration with diabetes was 11.7 ± 11.0 years. Demographic, anthropometric, and clinical data at baseline are listed in Table 1. One hundred and eighty-nine patients (14.7%) received no medication; 781 (60.9%) engaged in habitual exercise, such as walking in leisure time; and 174 had a history of CVD (13.6%). The number of patients with sarcopenia was 369 (28.8%).

**Table 1:** Clinical characteristics of the subjects.

	All	Men	Women
**Demographics**			
n	1282	709	573
Age (years)	63.8 (13.9)	63 (14.2)	64.8 (13.4)
Alcohol consumption (g ethanol per day)	18.8 (32)	26.8 (36.8)	9 (22.9)
Smoking habit (yes/no)	327/955	219/490	108/465
Brinkman index	328 (549)	328 (549)	123 (300)
Habitual exercise (yes/no)	781/501	440/269	341/232
Exercise time (min/day)	16 (45.9)	18 (54)	13.6 (33.2)
Locomotive regular exercise (MET-hours/week)	20.5 (20.2)	22.1 (22.9)	18.3 (15.6)
History of cardiovascular disease	174	111	63
Stroke	91	60	31
Myocardial infarction	92	56	36
Peripheral artery disease	10	8	2
Duration of diabetes (years)	11.7 (11)	11.8 (11)	11.6 (11.1)
Medication (yes/no)	1093/189	609/100	484/89
**Anthropometric data**			
Height (cm)	160.7 (9.6)	167.1 (6.7)	152.8 (6.2)
Weight (kg)	66.2 (16.8)	70.8 (17.1)	60.5 (14.7)
BMI (kg/m^2^)	25.5 (5.4)	25.2 (5.1)	25.8 (5.8)
Waist circumference (cm)	92 (13.7)	92 (13.5)	91.9 (14.1)
Handgrip strength (kg)	24 (9.7)	29.2 (8.7)	17.5 (6.4)
**Body composition** (**n = 122**)			
Skeletal muscle mass (kg)	25.3 (6.3)	28.8 (5.7)	21 (3.7)
Body fat mass (kg)	24.2 (12.9)	22.6 (13.1)	26.1 (12.5)
Body fat percentage (%)	32.5 (10.9)	28.2 (9.5)	37.7 (10.2)
**Physiological and biochemical data**			
Systolic blood pressure (mmHg)	131.1 (19.8)	132.6 (19.4)	129.2 (20.2)
Diastolic blood pressure (mmHg)	73.6 (14.1)	76.1 (13.7)	70.4 (14.1)
Plasma glucose (mg/dL)	159 (63.8)	163 (65.7)	154.1 (61.1)
HbA1c (%)	7.5 (1.7)	7.6 (1.8)	7.4 (1.6)
Serum insulin (μU/mL) (n = 209)	9.9 (8.3)	9.6 (7.7)	10.4 (9)
Serum C-peptide (ng/mL) (n = 553)	2.5 (2)	2.5 (1.9)	2.6 (2.2)
Estimated glomerular filtration rate (mL/min/1.73 m^2^)	72.9 (23.5)	73.2 (23.4)	72.5 (23.7)
Urinary albumin creatinine ratio (mg/gCr)	146.9 (539.1)	183 (667.6)	98.7 (284.4)
Brachial-ankle pulse wave velocity (cm/s)	1713.9 (407.6)	1728.5 (405.7)	1695.7 (409.7)
Augmentation index	75.6 (13.1)	72 (13.4)	81.1 (10.7)

Data are represented as the mean (SD) except for the number of subjects and sex. BMI: body mass index, HbA1c: hemoglobin A1c.

Handgrip strength was inversely correlated with age, duration of diabetes, fat mass percentage, brachial-ankle pulse wave velocity (baPWV), augmentation index (AIx_75_), and the number of hospitalizations. In contrast, handgrip strength was positively correlated with BMI, waist circumference, alcohol consumption, exercise time, walking time, amount of locomotive exercise, skeletal muscle mass, systolic and diastolic blood pressure, estimated glomerular filtration rate (eGFR), and duration before hospitalization. Similar correlations were observed in both men and women; however, there was no significant correlation of handgrip strength with walking time, locomotive regular exercise, fat mass percentage, and systolic blood pressure in men and with exercise time, fat mass percentage, and systolic blood pressure in women (Table 2). After adjustment for age, sex, and BMI, handgrip strength was still inversely associated with waist circumference, plasma glucose (PG) levels, serum C-peptide levels, urinary albumin creatinine ratio (UACR), fat mass percentage, and the number of hospitalizations, whereas it was positively associated with alcohol consumption, diastolic blood pressure, exercise time, walking time, amount of locomotive exercise, skeletal muscle mass, and duration before the first hospitalization after the measurement of handgrip strength. Moreover, handgrip strength was positively associated with eGFR after adjustment for BMI (Supplementary Table 1).

**Table 2:** Correlations between handgrip strength and clinical parameters.

	All		Men		Women	
	r	P	r	r	r	P
Age	−0.394	<0.001	−0.465	<0.001	−0.413	<0.001
BMI	0.156	<0.001	0.291	<0.001	0.163	<0.001
Waist circumference	0.124	<0.001	0.202	<0.001	0.075	0.078
Exercise time	0.087	0.002	0.081	0.033	0.054	0.21
Walking time	0.127	<0.001	0.046	0.23	0.196	<0.001
Locomotive regular exercise	0.133	0.001	0.067	0.2	0.183	0.003
Skeletal muscle mass	0.679	<0.001	0.499	<0.001	0.395	0.003
Fat mass percentage	−0.219	0.015	0.19	0.12	−0.075	0.59
Systolic blood pressure	0.061	0.029	0.008	0.84	0.019	0.66
Diastolic blood pressure	0.283	<0.001	0.247	<0.001	0.15	<0.001
Plasma glucose	−0.017	0.55	−0.066	0.082	−0.086	0.043
HbA1c	0.039	0.17	0.039	0.31	−0.012	0.78
Serum C-peptide	−0.044	0.31	−0.038	0.53	−0.042	0.49
Estimated glomerular filtration rate	0.124	<0.001	0.145	<0.001	0.15	<0.001
Urinary albumin creatinine ratio	−0.008	0.85	−0.102	0.072	0.0042	0.52
baPWV	−0.2	<0.001	−0.298	<0.001	−0.248	<0.001
AIx_75_	−0.372	<0.001	−0.264	<0.001	−0.152	0.051
Alcohol consumption	0.24	<0.001	0.092	0.017	0.121	0.004
Duration of diabetes	−0.118	<0.001	−0.149	<0.001	−0.165	<0.001
Duration before hospitalization	0.103	<0.001	0.174	<0.001	0.104	0.014
Number of hospitalizations	−0.07	0.012	−0.105	0.005	−0.128	0.002

BMI: body mass index, HbA1c: hemoglobin A1c, baPWV: brachial-ankle pulse wave velocity, AIx_75_: augmentation index.

During a mean follow-up of 2.36 ± 0.73 years, 20 patients (1.6%) died, 14 (1.1%) experienced CVD events, and 556 (43.4%) were admitted to our hospital for any disease. Cox proportional hazards analyses with adjustment for age, sex, BMI, smoking habit, alcohol consumption, exercise time, eGFR, HbA1c, duration of diabetes, and medications, such as antihypertensive agents and glucose lowering agents, revealed that handgrip strength had a significant association with occurrence of CVD events [hazard ratio (HR) = 0.899; 95% confidence interval (CI), 0.819–0.987; p = 0.025] and hospitalization (HR = 0.964; 95% CI, 0.951–0.977; p < 0.001) (Table 3). Although the estimates were imprecise, similar patterns were observed in sex-stratified analyses. Specifically, handgrip strength was associated with mortality (HR = 0.899; 95% CI, 0.814–0.993; p = 0.037) and hospitalization (HR = 0.962; 95% CI, 0.946–0.977; p < 0.001) in men (Table 4) and with hospitalization in women (HR = 0.972; 95% CI, 0.948–0.991; p = 0.031) (Table 5).

**Table 3:** Cox proportional hazard analysis for evaluating the associations of handgrip strength with all–cause mortality, CVD events, and hospitalization in patients with type 2 diabetes.

	**All-cause mortality**			**CVD events**			**Hospitalization**		
	HR	95% CI	P	HR	95% CI	P	HR	95% CI	P
Age (per 1 year increase)	0.983	0.930–1.039	0.54	1.079	1.005–1.158	0.036	1.015	1.005–1.025	0.004
**Sex**									
Male	0.987	0.220–4.437	0.99	6.078	1.251–29.527	0.025	1.765	1.373–2.270	<0.001
Female	(ref)			(ref)			(ref)		
BMI (per 1 unit increase in kg/m^2^)	0.821	0.717–0.939	0.04	1.181	1.048–1.331	0.006	1.029	1.009–1.049	0.004
Smoking (per 1 unit increase in Brinkman index)	1.001	1.001–1.002	<0.001	0.999	0.997–1.001	0.21	1.000	1.000–1.000	0.29
Alcohol consumption (per 1 g/day increase in ethanol consumption)	1.308	1.095–1.563	0.003	0.986	0.566–1.715	0.96	1.060	1.004–1.118	0.034
**Medications**									
No medication	0.000	0.000	0.98	0.393	0.078–1.978	0.26	0.799	0.609–1.050	0.11
With medication	(ref)			(ref)			(ref)		
Exercise time (per 1 min/day increase)	0.997	0953–1.010	0.7	1.006	0.998–1.015	0.14	0.998	0.996–1.000	0.12
Duration of diabetes (per 1 year increase)	1.018	0.975–1.063	0.42	1.033	0.989–1.079	0.15	1.002	0.994–1.011	0.59
eGFR (per 1 unit increase in mL/min/1.73 m^2^)	0.977	0.953–1.000	0.051	1.016	0.985–1.047	0.32	1.001	0.996–1.005	0.8
HbA1c (per 1% increase)	1.226	0.868–1.730	0.25	0.904	0.601–1.359	0.63	1.364	1.295–1.437	<0.001
Handgrip strength (per 1 kg increase)	0.926	0.865–1.003	0.058	0.899	0.819–0.987	0.025	0.964	0.951–0.977	<0.001

CVD: cardiovascular disease, HR: hazard ratio, CI: confidence interval, ref: reference.

**Table 4:** Cox proportional hazard analysis for evaluating the associations of handgrip strength with all-cause mortality, CVD events, and hospitalization in male patients with type 2 diabetes.

	All-cause mortality			CVD events			Hospitalization		
	HR	95% CI	P	HR	95% CI	P	HR	95% CI	P
Age (per 1 year increase)	0.970	0.899–1.048	0.45	1.098	0.991–1.216	0.073	1.015	1.002–1.029	0.025
BMI (per 1 unit increase in kg/m^2^)	0.790	0.649–0.961	0.018	1.196	1.009–1.419	0.04	1.018	0.989–1.048	0.22
Smoking (per 1 unit increase in Brinkman index:)	1.001	1.000–1.002	0.004	0.999	0.997–1.001	0.21	1.000	1.000–1.000	0.84
Alcohol consumption (per 1 g/day increase in ethanol consumption)	1.278	1.050–1.557	0.015	0.966	0.502–1.855	0.92	1.051	0.988–1.119	0.12
**Medications**									
No medication	0.000	0.000	0.98	0.000	0.000	0.99	1.335	0.936–1.904	0.11
With medication	(ref)			(ref)			(ref)		
Exercise time (per 1 min/day increase)	0.995	0975–1.014	0.59	0.996	0.972–1.021	0.76	0.999	0.997–1.001	0.32
Duration of diabetes (per 1 year increase)	1.044	0.996–1.094	0.072	1.029	0.977–1.083	0.28	1.000	0.990–1.011	0.94
eGFR (per 1 unit increase in mL/min/1.73 m^2^)	0.986	0.957–1.015	0.34	1.016	0.974–1.060	0.47	1.003	0.997–1.009	0.37
HbA1c (per 1% increase)	1.319	0.865–2.012	0.2	1.103	0.674–1.805	0.7	1.330	1.243–1.422	<0.001
Handgrip strength (per 1 kg increase)	0.899	0.814–0.993	0.037	0.924	0.820–1.041	0.19	0.962	0.946–0.977	<0.001

CVD: cardiovascular disease, HR: hazard ratio, CI: confidence interval, ref: reference.

**Table 5:** Cox proportional hazard analysis for evaluating the associations of handgrip strength with all–cause mortality, CVD events, and hospitalization in female patients with type 2 diabetes.

	All-cause mortality			CVD events			Hospitalization		
	HR	95% CI	P	HR	95% CI	P	HR	95% CI	P
Age (per 1 year increase)	1.010	0.907–1.125	0.86	1.055	0.951–1.171	0.31	1.016	1.001–1.031	0.04
BMI (per 1 unit increase in kg/m^2^)	0.771	0.565–1.051	0.1	1.158	0.954–1.406	0.14	1.036	1.009–1.064	0.01
Smoking (per 1 unit increase in Brinkman index)	1.002	1.001–1.004	0.001	1.000	0.996–1.005	0.96	1.001	1.000–1.001	0.002
Alcohol consumption (per 1 g/day increase in ethanol consumption)	1.109	0.452–2.724	0.82	0.959	0.380–2.422	0.93	1.069	0.952–1.199	0.26
**Medications**									
No medication	0.000	0.000	0.98	21.781	2.020–234.835	0.011	1.149	0.740–1.785	0.54
With medication	(ref)			(ref)			(ref)		
Exercise time (per 1 min/day increase)	1.007	0987–1.029	0.49	1.024	1.007–1.041	0.006	0.996	0.990–1.001	0.11
Duration of diabetes (per 1 year increase)	0.924	0.822–1.039	0.19	1.055	0.971–1.146	0.21	1.006	0.991–1.020	0.44
eGFR (per 1 unit increase in mL/min/1.73 m^2^)	0.944	0.886–1.005	0.073	1.030	0.982–1.080	0.22	0.998	0.990–1.005	0.51
HbA1c (per 1% increase)	1.139	0.538–2.413	0.73	0.691	0.298–1.604	0.39	1.448	1.330–1.575	<0.001
Handgrip strength (per 1 kg increase)	0.921	0.762–1.112	0.39	0.846	0.703–1.018	0.077	0.972	0.948–0.997	0.031

CVD: cardiovascular disease, HR: hazard ratio, CI: confidence interval, ref: reference.

## Discussion

We demonstrated that handgrip strength was favorably associated with abdominal obesity renal function, and a marker of diabetic nephropathy in addition to being associated with occurrence of CVD events and hospitalization in Japanese patients with type 2 diabetes. In addition, handgrip strength was significantly associated with mortality and hospitalization in men and with hospitalization in women. To our knowledge, this is the first study to report significant associations between handgrip strength and prognosis in an Asian population with type 2 diabetes.

Handgrip strength has been identified as an indicator of mortality and CVD risk in healthy men^19, 22^, older individuals^20^, and prediabetic and diabetic patients^23^. A previous study showed that handgrip strength was associated with leisure-time physical activity^24^, which is known to be a protective factor against CVD and mortality in patients with type 2 diabetes^25^. Handgrip strength was positively associated with walking time and amount of locomotive exercise, which may contribute to the reduction in mortality and CVD risk^26^.

A few studies have investigated the relationship between handgrip strength and hospitalization^27, 28^. Cawthon *et al*.^27^ reported that older individuals with lower handgrip strength had an increased risk for hospitalization by 56% during a follow-up period of 4.7 years. Handgrip strength was also positively associated with duration before the first hospitalization, which suggested that handgrip strength could be an objective indicator for overall health.

Negative associations of handgrip strength with waist circumference and fat mass percentage indicate that muscular strength has a beneficial effect on obesity. Mason *et al*.^29^ reported that a low level of musculoskeletal fitness, including handgrip strength, was independently associated with higher weight gain. Jackson *et al*.^30^ also showed that muscular strength was inversely associated with the prevalence and incidence of abdominal obesity and excessive fat mass. These findings suggest that not only exercise but muscular strength could have a role in preventing obesity.

Other noteworthy findings of this study are the inverse association between handgrip strength and UACR and the positive association between handgrip strength and eGFR. Patients with chronic kidney disease (CKD) are exposed to the risk of skeletal muscle loss due to its comorbidities, complications, and treatments^31^. Thus, it is crucial for patients with CKD to assess muscle strength during an early stage and to prevent sarcopenia and frailty. Lattanzio *et al*.^32^ showed that eGFR was independently and positively associated with muscle strength in older hospitalized patients. The Korea National Health and Nutrition Examination Surveys of 2008–2011 reported that sarcopenia was independently associated with albuminuria (odds ratio = 1.61)^33^. These findings are consistent with our results. Although we could not deduce a causal relationship, maintaining muscle strength may preserve renal function in patients with type 2 diabetes. Handgrip strength has also been found to be a useful predictor of mortality in patients on maintenance dialysis^34, 35^ and renal outcome in patients with CKD^36^. Clinicians could use handgrip strength as an indicator of renal prognosis beyond mere muscle strength in patients with type 2 diabetes.

A previous review showed that there was a dose–response relationship between alcohol consumption and physical activity level^37^. In the present study, subjects consumed <20 g of ethanol on average, and we found a positive association between alcohol consumption and exercise time after adjustment for age, sex, and BMI (β = 0.059, P = 0.033). Handgrip strength was positively associated with alcohol consumption because individuals who consume moderate amounts of alcohol may be physically active and have greater muscle strength. In addition, alcohol consumption was associated with increasing energy intake, and current drinkers had significantly higher intakes of protein than never drinkers^38^. Intakes of nutrients involved in alcohol consumption may influence the handgrip strength. Grip strength was lower in individuals with hypertension than in those without^39^. However, we observed that diastolic blood pressure was positively associated with handgrip strength. After further adjustment for the use of antihypertensive drugs, diastolic blood pressure was still positively associated with handgrip strength. Recently, a few studies have reported a positive association between blood pressure and muscle strength in the elderly and in adolescents^40, 41^. Simple regression analysis showed that handgrip strength was inversely correlated with AIx_75_; however, it was more positively correlated with diastolic blood pressure than systolic blood pressure in the present study. Nürnberger *et al*. reported that not systolic but diastolic blood pressure is a strong determinant of augmentation index in normotensive subjects^42^. Ageing and type 2 diabetes advances arterial stiffness and decreases diastolic blood pressure^43, 44^. Insulin resistance in type 2 diabetes causes delay in vasodilation^45^. Augmentation index, a measure of wave reflection, which is modulated by arterial elasticity and peripheral vascular resistance, is paradoxically lower, and diastolic blood pressure is also lower in patients with diabetes than in those without diabetes^46^. Handgrip exercise decreases vascular resistance via a cholinergic mechanism in healthy humans^47^; however, such a physiological effect may be attenuated in type 2 diabetic patients with autonomic nervous dysfunction. The underlying mechanism was not clear; however, the sympathoexcitatory effect of insulin may explain this result^48^. Muscle sympathetic nerve activity increases in response to hyperinsulinemia, which increases peripheral vascular resistance and leads to high blood pressure^40, 48^. In fact, we found a positive association between diastolic blood pressure and serum C peptide levels after adjustment for age and sex. However, to elucidate the mechanism of association between handgrip strength and blood pressure, further investigations are required.

This study had several limitations. First, although all subjects were instructed to follow a diet therapy for diabetes, we did not objectively evaluate dietary intake. To maintain muscle strength and mass, dietary intake of a certain amount of protein is needed. Dietary intake may affect handgrip strength. Second, we used consecutive sampling in order to avoid selection bias; however, lack of representativeness could occur. The present study was a single-center study, and the results cannot be generalized to other populations. Third, considering that the study subjects were not newly diagnosed with type 2 diabetes, handgrip strength at baseline could be influenced by many conditions. We performed Cox proportional hazards analysis after adjusting for potential confounding variables; however, residual confounding factors, including comorbidities and socioeconomic status, might have been associated with handgrip strength. In addition, other factors, such as time of clinical examination, that influence subjects’ condition and body composition analysis also need to be controlled. Fourth, the follow-up period was relatively short. Therefore, the number of deaths and CVD events was small. We should continue further investigations. Despite these limitations, we demonstrated that handgrip strength was significantly associated with various health indices in patients with type 2 diabetes.

In conclusion, the findings of this study suggest that handgrip strength could be a prognostic indicator for health and a diagnostic marker of sarcopenia in patients with type 2 diabetes. Measuring handgrip strength is quick and inexpensive. Handgrip strength could be a useful tool in the management of type 2 diabetes.

## Methods

### Study design and subjects

We conducted a retrospective cohort study in patients with type 2 diabetes who were treated at the National Center for Global Health and Medicine Kohnodai Hospital. Between April 2013 and December 2015, a total of 1,303 individuals whose handgrip strength was measured at the first medical examination were included. Patients younger than 20 years (n = 2) with type 1 diabetes (n = 16) were excluded. Patients whose handgrip strength could not be measured because of disabilities, such as cerebral infarction sequelae (n = 3), were also excluded. We conducted a post-hoc sample size calculation using the powerlog command in Stata (http://www.ats.ucla.edu/stat/sas/dae/logit_power.htm). Assuming the proportion of death at mean handgrip strength is 1.6%, the proportion of death at one standard deviation above the mean handgrip strength is 0.74%, and the one-tailed alpha level is 0.025, the sample size calculation indicates that 796 observations are needed for a power of 0.8. This suggests that our sample size had sufficient power to detect the observed association between handgrip strength and all-cause mortality. At the first visit, patients were instructed to consume a calorie-restricted diet of 25–30 kcal/kg (ideal body weight) per day as diet therapy for diabetes by certified nutritional educators and to continue the diet during study period. All patients were evaluated and followed until death or till the end of follow-up in May 2016. We retrieved data, such as date of hospital admission, duration of hospitalization, name of disease for hospitalization, date of occurrence of CVD, and date of death from the electronic health record in our hospital.

### Anthropometric and physiological measurements

Height was measured using a rigid stadiometer (TTM stadiometer; Tsutsumi Co., Ltd., Tokyo, Japan). Weight was measured using calibrated scales (AD-6107NW; A&D Medical Co., Ltd., Tokyo, Japan). BMI was calculated as body weight in kilograms divided by the square of body height in meters. Waist circumference was measured at the umbilical level at the end of exhalation in a standing position. Handgrip strength was measured using a Smedley analog hand dynamometer (No. 04125; MIS, Tokyo, Japan) with both hands in a standing position. The measurements were first performed for the dominant hand. Subjects performed two maximum attempts for each hand with approximately 1-min rest period between trials. We used the average handgrip strength in kilograms. According to a consensus report of the Asian Working Group for Sarcopenia, we defined sarcopenic subjects as follows: age, ≥ 65 years and handgrip strength, <26 kg for men and <18 kg for women^49^.

Blood pressure was measured in a seated position using an automatic sphygmomanometer (HBP-9020; Omron Co., Ltd, Tokyo, Japan). Arterial stiffness was examined by measuring both baPWV and AIx_75_ using a pulse pressure analyzer (BP-203RPE; Nihon Colin, Tokyo, Japan) and a digital automatic sphygmomanometer (HEM-9000AI; Omron Co., Ltd, Tokyo, Japan), respectively.

### History taking and physical activity assessment

For baseline characteristics, trained technicians at the Clinical Research Center of the National Center for Global Health and Medicine at Kohnodai Hospital asked participants at the outpatient clinic about their physical activity levels, smoking, drinking habits, medication, and history of CVD. CVD included stroke, nonfatal coronary heart disease, or peripheral artery disease. To quantify the patients’ smoking habits, the Brinkman index (number of cigarettes per day multiplied by the number of years) was calculated^50^. Patients were also asked about their regular exercise habits, and we estimated the amount of locomotive exercise using metabolic equivalent (MET) hours per week. Briefly, the physical activity questionnaire consists of 3 items: 1) “Do you engage in any physical exercise? If yes, what kind of exercise do you perform regularly?”; 2) “How often do you exercise in a week?”; and 3) “How long do you exercise per session?” We calculated MET-hours per week for locomotive physical exercise based on the Compendium of Physical Activities^51^.

### Blood and urinary examination

Blood samples were taken from the antecubital vein in the morning. We measured PG, hemoglobin A1c, insulin, and C-peptide levels. Enzymatic methods were used to assess PG (Glucose Assay Kit; Wako Pure Chemical Industries, Osaka, Japan). Serum insulin and HbA1c were measured by automated enzyme-linked immunosorbent assays (E-test TOSOH II; Tosoh, Tokyo, Japan) and high-performance liquid chromatography (HA-8180; Arkray, Tokyo, Japan), respectively. Serum C-peptide was measured using a chemiluminescent enzyme immunoassay (LUMIPULSE; Fujirebio, Tokyo, Japan). We calculated eGFR using the revised equation adjusted for the Japanese population^52^. UACR, a marker for diabetic nephropathy, was also measured. The urinary albumin levels were measured using an immunoturbidimetric assay (SRL, Tokyo, Japan), and UACR was calculated by dividing the urinary albumin levels by the urinary creatinine concentration.

### Body composition analysis

Body composition was analyzed using a bioelectrical impedance analysis device (InBody720; Biospace Co., Ltd, Tokyo, Japan). This method is based on the principle that lean body mass contains higher water and electrolyte content than fat tissue; hence, these tissues can be distinguished by electrical impedance. Body composition was determined using a patented 8-point tactile electrode system^53^. A previous validation study demonstrated that body composition measured using this device was highly correlated with dual-energy X-ray absorptiometric measurements^54^.

### Statistical analysis

Statistical analyses were performed using SPSS version 23 (IBM Co., Ltd., Chicago, IL). All values are expressed as the mean ± standard deviation except for sex and smoking habit. Pearson’s correlation coefficient was calculated to analyze the associations of handgrip strength with physical, biochemical, and physiological data in overall sample as well as in male and female subjects. Multiple regression analysis adjusted for age, sex, and BMI was performed to test independent associations between handgrip strength and clinical data. Furthermore, Cox proportional hazards analysis was performed to assess the independent associations of mortality, CVD events, and hospitalization. We included age, sex, BMI, smoking (Brinkman index), alcohol consumption, exercise time, eGFR, HbA1c, duration of diabetes, and medications such as antihypertensive agents and glucose-lowering agents in the Cox model. The entry variable was the date of the measurement of handgrip strength. Follow-up was censored at the first event, death, or May 1, 2016, whichever came first. We also performed Cox proportional hazards analysis stratified by sex because the difference in handgrip strength is large between men and women. In addition, p values of <0.05 determined by performing a two-sided test were considered to be statistically significant.

### Ethics Statement

Because this study was a retrospective cohort study, the opt-out method of obtaining informed consent was adopted. The patients were anonymized to protect their personal information. The study protocol was approved by the Medical Ethics Committee of the National Center for Global Health and Medicine (Reference No. NCGM-G-002052), and the study was performed in accordance with the Declaration of Helsinki.

### Data Availability

The datasets generated and/or analyzed during the current study are available from the corresponding author on reasonable request.

## Electronic supplementary material

Supplementary information
