# Supplementary material for: Association of handgrip strength with hospitalization, cardiovascular events, and mortality in Japanese patients with type 2 diabetes
Source: Sci Rep. 2017 Aug 1;7:7041. doi: 10.1038/s41598-017-07438-8 (PMC5539205; doi:10.1038/s41598-017-07438-8)
Supplement: Supplementary file 1 — Supplementary information [file 41598_2017_7438_MOESM1_ESM.pdf]

Association of handgrip strength with hospitalization, cardiovascular events, and mortality in Japanese patients with type 2 diabetes

Hidetaka Hamasaki[1,2,*], Yu Kawashima[1], Hisayuki Katsuyama[3], Akahito Sako[1], Atsushi Goto[4], and Hidekatsu Yanai[1]

[1]Department of Internal Medicine, National Center for Global Health and Medicine Kohnodai Hospital, 1-7-1 Kohnodai, Ichikawa, Chiba 272-8516, Japan

[2]Hamasaki Clinic, 2-21-4, Nishida, Kagoshima 890-0041, Japan

[3]Institute for Clinical Diabetology, German Diabetes Center, Leibniz Center for Diabetes Research, Heinrich Heine University, Auf'm Hennekamp 65, Düsseldorf 40225, Germany

[4]Epidemiology and Prevention Group, Center for Public Health Sciences, National Cancer Center, 5-1-1 Tsukiji, Chuo-ku, Tokyo 104-0045, Japan

*Corresponding author: Hidetaka Hamasaki

Hamasaki Clinic, 2-21-4, Nishida, Kagoshima 890-0041, Japan

Tel: +81-99-2503535; Fax: +81-99-2501470

E-mail address: hhamasaki78@gmail.com (H. Hamasaki)

	All		Men		Women	
	**β**	**P**	**β**	**P**	**β**	**P**
Waist circumference	−0.103	0.012	−0.219	0.003	−0.096	0.186
Exercise time	0.085	<0.001	0.105	0.002	0.102	0.009
Walking time	0.076	<0.001	0.062	0.068	0.185	<0.001
Locomotive regular exercise	0.072	0.016	0.064	0.175	0.162	0.005
Skeletal muscle mass	0.507	<0.001	0.416	0.012	0.665	0.001
Fat mass percentage	−0.344	0.015	−0.131	0.579	−0.707	0.003
Systolic blood pressure	0.013	0.53	-0.028	0.433	0.060	0.13

Diastolic blood pressure	0.057	0.008	0.077	0.032	0.05	0.21
Plasma glucose	−0.068	0.001	−0.063	0.059	−0.112	0.004
HbA1c	−0.024	0.23	−0.028	0.4	−0.042	0.28
Serum C-peptide	−0.074	0.024	−0.095	0.097	−0.092	0.12
Estimated glomerular filtration rate	0.121	<0.001	0.146	<0.001	0.142	0.001
Urinary albumin creatinine ratio	−0.073	0.021	−0.131	0.011	0.044	0.47
Alcohol consumption	0.058	0.006	0.094	0.006	0.043	0.28
Duration before hospitalization	0.103	<0.001	0.155	<0.001	0.085	0.029

Number of hospitalizations	−0.069	0.001	−0.070	0.035	−0.112	0.004

Supplementary Table 1. Associations between handgrip strength and clinical parameters

The multiple regression models were adjusted for age, sex, and body mass index in all subjects, and adjusted for age and body mass index in men and women. HbA1c: hemoglobin A1c
